# Top-Down Influences on Local Networks: Basic Theory with Experimental Implications

**DOI:** 10.3389/fncom.2013.00029

**Published:** 2013-04-18

**Authors:** Ramesh Srinivasan, Samuel Thorpe, Paul L. Nunez

**Affiliations:** ^1^Department of Cognitive Sciences, University of CaliforniaIrvine, CA, USA; ^2^Department of Biomedical Engineering, University of CaliforniaIrvine, CA, USA; ^3^Institute for Mathematical Behavioral Sciences, University of CaliforniaIrvine, CA, USA; ^4^Cognitive Dissonance, LLCSan Diego, CA, USA

**Keywords:** EEG, population dynamics, neural mass models, top-down control, ECoG

## Abstract

The response of a population of cortical neurons to an external stimulus depends not only on the receptive field properties of the neurons, but also the level of arousal and attention or goal-oriented cognitive biases that guide information processing. These top-down effects on cortical neurons bias the output of the neurons and affect behavioral outcomes such as stimulus detection, discrimination, and response time. In any physiological study, neural dynamics are observed in a specific brain state; the background state partly determines neuronal excitability. Experimental studies in humans and animal models have also demonstrated that slow oscillations (typically in the alpha or theta bands) modulate the fast oscillations (gamma band) associated with local networks of neurons. Cross-frequency interaction is of interest as a mechanism for top-down or bottom up interactions between systems at different spatial scales. We develop a generic model of top-down influences on local networks appropriate for comparison with EEG. EEG provides excellent temporal resolution to investigate neuronal oscillations but is space-averaged on the cm scale. Thus, appropriate EEG models are developed in terms of population synaptic activity. We used the Wilson–Cowan population model to investigate fast (gamma band) oscillations generated by a local network of excitatory and inhibitory neurons. We modified the Wilson–Cowan equations to make them more physiologically realistic by explicitly incorporating background state variables into the model. We found that the population response is strongly influenced by the background state. We apply the model to reproduce the modulation of gamma rhythms by theta rhythms as has been observed in animal models and human ECoG and EEG studies. The concept of a dynamic background state presented here using the Wilson–Cowan model can be readily applied to incorporate top-down modulation in more detailed models of specific cortical systems.

## Introduction

A fundamental question in any neurophysiological study is whether observed modulations of neural responses in cortex by cognitive processes are the result of the action of a *local* network or due to the interactions between this local network and the rest of the brain in *global* networks. This conceptual framework of local and global networks interacting in cognitive processes is salient to the interpretation of physiological signals obtained from the brain with any technique – EEG, MEG, fMRI, LFPs, or unit activity and to models of the underlying cognitive processes. That is, even when signals are recorded from a small number of neurons (or even just one neuron) the observed dynamics result both from the intrinsic properties of the local network and from the influence of other neurons located in nearby or even distant cortex (Mountcastle, 1997). This simple distinction can be understood in terms of behavior – the response of neurons to inputs depends not only on the receptive field of the neurons but also on the level of arousal, typically by the action of neuromodulators, and attention or goal-oriented cognitive biases that guide information processing. The latter are sometimes called top-down effects (Engel et al., 2001), which bias the output of the neurons and affect behavioral outcomes such as stimulus detection, short term memory, and reaction time.

The objective of this paper is to develop a model of local networks with which we can investigate the effect background brain state or top-down signaling on the local network. In order to develop this model, we have to make choices of spatial scale and physiological detail to incorporate into the model. Very detailed models have the potential to provide more information about specific neural systems, e.g., details models of the visual system (Lumer et al., 1997). However, detailed model parameters are not available in humans, where the competition/interaction between global and local dynamics is expected to be the most robust (Nunez, 1995, 2000; Nunez and Srinivasan, 2006). In addition, detailed models may not lead to generalized principles that can potentially guide experimental studies in a variety of behavioral contexts; detailed cellular models are not easily compared to electrocorticogram (ECoG) and electroencephalogram (EEG) data obtained in humans.

Electroencephalogram is uniquely positioned to differentiate local and global processes and to examine their interactions in human subjects. EEG provides excellent temporal resolution allowing us to separate processes at different time scales at electrodes over cortex while allowing for sufficient spatial coverage to investigate interactions of sensory neurons with neural processes in other areas of the brain. The main limitation of EEG is spatial resolution; EEG signals are space-averaged on the cm scale (Nunez, 1981; Nunez and Srinivasan, 2006) by volume conduction through the tissues of the head. An active area of research is to improve our understanding of the structure of cortical sources and connectivity from EEG data (Pinotsis et al., 2012). ECoG in humans combines the temporal dynamics of EEG with the greater spatial detail and (depending on the patient) partial coverage of the cortex (Schalk and Leuthardt, 2011). Although ECoG is only available in limited cases in patients with intractable epilepsy, these data are a useful source of information on the nature of dynamics of localized population of neurons.

EEG signals span a frequency range of 1–50 Hz while ECoG signals span a broader frequency range of 1–150 Hz (Canolty et al., 2010; Schalk and Leuthardt, 2011). The lower portion of this spectrum (below 20 Hz) has strongly global properties with spatial distribution across the brain that depends strongly on the frequency (von Stein and Sarnthein, 2000; Nunez et al., 2001; Nunez and Srinivasan, 2006). For example human alpha rhythms, which are quite robust in alert subjects, may be recorded over nearly all of the upper scalp or cortex with a visible peak in the power spectrum near 10 Hz. Alpha rhythm power and phase synchronization (usually measured as coherence) are modulated in specific large-scale cortical networks by a wide variety of different cognitive processes including attention (Thut et al., 2006; Thorpe et al., 2012) and working memory (Sarnthein et al., 1998; Sauseng et al., 2005). Consistent with this “global” picture of low frequency EEG signals are studies using periodic visual input to elicit steady-state visual evoked potentials (SSVEPs). SSVEPs are responses to visual flicker at the flicker frequency (and harmonics). Low frequency (<20 Hz) SSVEPs elicit “resonant” responses in large-scale networks whose spatial distribution depends strongly on the input temporal frequency (Ding et al., 2006; Srinivasan et al., 2006). These large-scale networks have both distinct characteristic frequencies and functional properties (Ding et al., 2006; Bridwell and Srinivasan, 2012).

At higher frequencies (>30 Hz) the spatial distribution of EEG and ECoG signals is (apparently) localized at the cm scale. EEG studies have shown task dependent modulations of gamma networks in networks localized in sensory and motor cortex. These studies were inspired by single-unit and LFP studies in animal models, most notably by Singer and colleagues (Engel and Singer, 2001; Fries et al., 2007) that demonstrate localized networks synchronizing at gamma band frequencies. This local view of the origin of gamma rhythms is supported by ECoG studies that show relatively low coherence between electrodes at gamma band frequencies (Menon et al., 1996). SSVEP data at gamma band frequencies are consistent with this localized picture of fast EEG rhythms – γ-SSVEPs appear to be local processes in the visual cortex (Thorpe et al., 2011).

The distinct spatial and dynamical property of EEG oscillations in low (<20 Hz) and high (>20 Hz) frequency bands suggests the need for different types of models to explain these phenomena. Given any unknown physical or biological system that produces oscillations at some preferred (or resonant) frequency *f* = ω/2π, a reasonable starting point for developing a model is the origin of the implied underlying time delay τ roughly estimated as

(1) $$\tau \sim \omega^{-1}$$

The implied physiological time scale for the (8–13 Hz) alpha rhythm is τ = 12–20 ms. More generally, the most robust human EEG rhythms recorded from the scalp (1–20 Hz) correspond to time delays τ = 8–160 ms. How does this delay range compare with mammalian physiology? Whereas early studies of membrane time constants in mammalian cortex were very short, typically less than 10 ms, more modern studies with improved recording methods report a wider range up to 100 ms (Koch et al., 1996). While synaptic delays (PSP rise and decay times) lie in a general range (within a factor of perhaps 5 or 10) that might account for dominant EEG frequencies, claims of close agreement between the *details* of observed EEG spectra and dynamic theories based on membrane time constants are not by themselves a critical validation of a model. Model parameters can always be chosen to “match” EEG data, which, in any case, varies widely between brain states.

*Local network* theories refers to models of cortical or thalamocortical interactions in which signal propagation delays in axons are neglected. For example, coupled non-linear oscillators interact without any transmission delay in a local theory. In contrast, models that incorporate the spatial extent of the cortex and the transmission delays between neural populations are *global* theories. Global theories predict spatially coherent oscillations over the surface of the cortex with wave-like properties that depend primarily on the transmission delays between cortical populations and the size (surface area) of the cortex (Nunez, 1981, 1995, 2000). The dominant modes of these spatially distributed oscillations are predicted to lie below 15 Hz in the theta and alpha bands. While both global and local network theories have been developed independently, their interaction across spatial and temporal scales is less well understood. Previous studies have focused on how local networks influence global networks (Jirsa and Haken, 1996; Nunez, 2000), and a recent study investigates the interaction between local connectivity and long-range interactions (Pinotsis et al., 2013). In this paper we consider how global network dynamics may influence local networks.

The underlying time scales in local network theories are typically postsynaptic potential rise and decay times due to membrane capacitive-resistive properties (Wilson and Cowan, 1972, 1973). Local theories typically predict EEG signals with frequencies above 20 Hz. These results are consistent with more detailed studies of spiking neuron models (Izhikevich, 2006; Izhikevich and Edelman, 2008) that predict fast frequency oscillations in cortical populations unless coupled with delays as in a global network. Physiologically realistic compartment models incorporating the interactions between excitatory and inhibitory populations in cortex give rise to fast oscillations at gamma band frequencies (Bush and Sejnowski, 1996; Traub et al., 1997; Whittington et al., 2000). In these types of model, the dynamics are determined primarily by the synaptic rise and decay times and the strength of excitatory and inhibitory synaptic connections. More specific local models in sensory systems incorporate the essential spiking dynamics and connectivity of thalamocortical networks (Lumer et al., 1997) also giving rise to gamma band oscillations. While the physiological detailed models are useful to compare to data in animal models, comparisons to EEG and ECoG require model development in macroscopic variables that describe synaptic mass action.

The Wilson–Cowan model is one of the earliest and most often cited dynamic models based on local (PSP rise and decay) delays (Wilson and Cowan, 1972, 1973). The Wilson–Cowan model produces either sustained (limit cycle) or damped oscillations over a broad range of physiologically realistic parameter space in response to a step function input to the excitatory population. The oscillations in all parts of the network are highly correlated, as there is no independent noise in each population. The rate of damping of the oscillations is largely determined by the ratio of excitatory to inhibitory weights with higher inhibition leading to damped oscillations. The frequency of the oscillation is determined primarily by the membrane time constants and connectivity strength.

In this paper, we will make use of the Wilson–Cowan model to investigate how properties of high frequency (gamma band) oscillations generated by a local network in response to input is influenced by modulation of the background state by top-down influences. Our objective here is to formalize the general principles by which local networks in cortex are influenced by modulatory signals. For this purpose, we have modified the Wilson–Cowan equations to make more physiologically realistic by incorporating background state parameters into the model. In any physiological study neural dynamics are observed in a specific brain state (e.g., asleep, awake, alert, attentive, etc.) determined partly by neuromodulatory action at much longer time scales. As brain state changes, the background state partly determines the excitability of the network (Fellous and Linster, 1998; Romei et al., 2008). Experimental studies in humans and animal models have also demonstrated that top-down influences in cognitive processes involve the action of slower oscillations typically in the alpha or theta bands which appear to reflect the coherent behavior of global networks distributed across the cortex. We believe that the mostly likely underlying time scale for such global oscillations is transmission delays in corticocortical axons, and we have proposed a specific global model that predicts global standing waves with frequencies in the general range at the slower end of the EEG spectrum (Nunez, 1995, 2000; Nunez and Srinivasan, 2006). Our analysis here depends only on the existence of such global, low frequency oscillations as has been commonly observed for almost 100 years with scalp EEG and not any specific global field theory of EEG. Using the modified Wilson–Cowan model, we identify cross-frequency coupling as an EEG or ECoG signature of the effects of background state changes by top-down signals on local network dynamics.

## Materials and Methods

### The modified Wilson–Cowan model

Wilson and Cowan (1972) derived a model neural population containing both excitatory and inhibitory neurons with dynamics described by a set of coupled, non-linear differential equations, herein labeled WC. The solution of these equations gives the proportion of cells in each subpopulation (excitatory/inhibitory) that become active per unit time. The cells comprising the population are assumed to be in close spatial proximity, with interconnections dense enough so that any two cells within it are path-connected. Furthermore, the model assumes that local interactions between neurons within the population are largely random, but that this local randomness gives rise to structure at larger spatial scales. The situation is analogous to an example taken from thermodynamics, in which a fluid with a macroscopically structured flow can be observed to be undergoing stochastic Brownian motion at the molecular level. The same framework set forth by Wilson and Cowan has been extended in a number of straightforward ways to models with more general connectivity, and an arbitrary number of spatially distinct neural populations (Campbell and Wang, 1996; Borisyuk et al., 2000). Extensions of the WC framework have been developed to model interacting thalamic (reticular formation) and cortical structures involved in the generation of spindle oscillations (7–14 Hz) in early sleep stages (Yousif and Denham, 2005). Jirsa and Haken (1997), used a WC model interacting with a global model to interpret MEG data in a syncopated tapping audio-motor task. Other model developments related to the WC model have incorporated spatially extended models with axonal delays and more detailed physiological parameters (Jirsa and Haken, 1996, 1997; Robinson et al., 1997; Liley et al., 1999).

**Figure 1:**
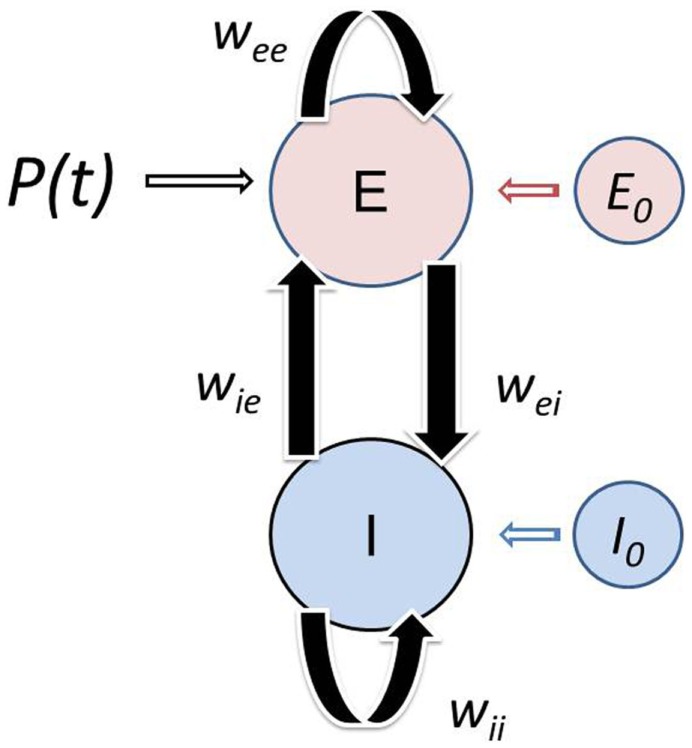
**Schematic of the modified Wilson–Cowan model**. The localized population consists of excitatory and inhibitory neurons that interact with each other with negligible transmission delays. The population receives afferent input *P*(*t*). Simultaneously, the population is subject to influences from both nearby and distant cortex. This top-down modulation of the neural population is the result of feedback from large-scale networks and/or global synaptic fields spanning the cortex. The model consists of an Excitatory (*E*) and Inhibitory (*I*) subpopulations with membrane time constants τ_*E*_ and τ_*I*_, which interact with each other via the connection weights *w_EI_* and *w_IE_*. The neurons within each subpopulation also interact with each other, reflected in the self-excitation *w_EE_* and self-inhibition *w_II_* weights. The influence of other cortical areas on the population is reflected in the background state of the excitatory *E*_0_ and inhibitory *I*_0_ subpopulation. The Wilson–Cowan model was modified to incorporate the background state variables (see Appendix).

Here we adopt a modified version of WC to make it more physiologically realistic as outlined in the Appendix. The basic dependent variables are the *fractions of excitatory and inhibitory active cells* (action potential densities) *E*(*t*), *I*(*t*), which can evidently exhibit high frequency jitter not treated in this analysis. Rather, the WC equations are expressed in terms of coarse grained excitatory 〈*E*(*t*)〉 and inhibitory 〈*I*(*t*)〉 action potential densities. The basic model is illustrated in Figure 1. We introduce the new dependent variables *X_E_*(*t*), *X_I_*(*t*), which provide perturbations about the critical (equilibrium) point (*E*_0_, *I*_0_), which we have interpreted as the background brain state which is controlled by various neuromodulators or top-down signaling. Thus, we express

(2) $$\begin{array}{rcll} \left\langle E\left(t\right)\right\rangle & =E_{0}+X_{E}\left(t\right) & & \\ \left\langle I\left(t\right)\right\rangle & =I_{0}+X_{I}\left(t\right) & \end{array}$$

Since the excitatory action potential densities are defined as fractions of the total cell populations, we require

(3) $$\begin{array}{rcll} 0 & \leq E_{0}+X_{E}\left(t\right)\leq 1 & & \\ 0 & \leq I_{0}+X_{I}\left(t\right)\leq 1 & \end{array}$$

The basic WC equations then become

(4) $$\begin{array}{rcll} \frac{dX_{E}}{dt} & =-E_{0}-X_{E}+\left(1-E_{0}-X_{E}\right)S_{E}\left(X_{E},X_{I},P\right) & & \\ A\frac{dX_{I}}{dt} & =-I_{0}-X_{I}+\left(1-I_{0}-X_{I}\right)S_{I}\left(X_{E},X_{I}\right) & \end{array}$$

Here $A=\frac{\tau_{I}}{\tau_{E}}$ is the ratio of inhibitory to excitatory time constants, and *P*(*t*) is an excitatory external (driving) input from another cortical population or potentially input to the population from the thalamus. The set of parameters (*w_EE_*, *w_IE_*, *w_EI_*, *w_II_*) are gain parameters that give the strength of connections between the excitatory and inhibitory populations as indicated in Figure 1. As shown in the Appendix for the special case *P*(*t*) = 0, the sigmoid functions *S_E_*, *S_I_* in Eq. 4 then take the forms

(5) $$\begin{array}{rcl} S_{E} & =\frac{1}{1+\left(\frac{1}{E_{0}}-2\right)\exp\left(-w_{EE}X_{E}+w_{IE}X_{I}-P\right)}\;E_{0}<\frac{1}{2} & \end{array}$$

(6) $$\begin{array}{rcl} S_{I} & =\frac{1}{1+\left(\frac{1}{I_{0}}-2\right)\exp\left(-w_{EI}X_{E}+w_{II}X_{I}\right)}\;I_{0}<\frac{1}{2} & \end{array}$$

### Parameter choices

For our simulations the main parameters of interest are the background state variables *E*_0_ and *I*_0_, which we will vary as described in the following sections. The free parameters in our analysis are the set of connection weights (*w_EE_*, *w_EI_*, *w_IE_*, *w_II_*) which are determined by the following physiological considerations: (1) In the cortex, excitatory connections are estimated to be 4–5 times more common than inhibitory connections (Bush and Sejnowski, 1996) and (2) Inhibitory connections are more typically found on the cell body possibly increasing their effectiveness in comparison to excitatory connections on dendritic trees (Mountcastle, 1997). Taking these two points into consideration we first fixed the two parameters *w_EI_* = 50 and *w_IE_* = 15. We set the self-inhibition *w_II_* = 0, as we found little practical effect for the small values of this parameter, other than to increase damping in the system, and shift the critical point for transition from a damped oscillation to a limit cycle regime.

Equation 4 produce stable limit cycle solutions about the critical point (*E*_0_, *I*_0_) for a wide range of the parameters. For example, setting *A* = 1 and *E*_0_ = *I*_0_ the necessary condition for oscillatory solutions about *E*_0_, *I*_0_ is

(7) $$w_{EE}<2\sqrt{w_{IE}w_{EI}}$$

This oscillatory solution is unstable (e.g., an unstable spiral allowing for a stable limit cycle) if

(8) $$w_{EE}>\frac{2}{E_{0}\left(1-2E_{0}\right)}$$

From Eq. 8 we were always able to find the critical value of *w*_EE_ below which the system produced damped oscillations in response to a step function input, while above this value the system produced limit cycle oscillations.

### Top-down (global) influences on a local WC network

We explicitly consider two types of top-down influences on the local WC network developed in section “The Modified Wilson–Cowan Model”: (1) the effect of neuromodulators setting the background state (*E*_0_, *I*_0_) of the population. For the purpose of the analysis here we consider this effect on the background state to be static as it takes place at very long time scales as compared to the frequency of the oscillations and (2) the effect of dynamic modulation of the background state of the local network (top-down) by oscillations in larger scale networks that incorporate the cells that constitute the local network. For simplicity of analysis we presume that the larger scale networks (or global synaptic fields) generate oscillations at frequency ω_α_ that modulate the background state of the WC oscillator; that is

(9) $$\begin{array}{rcll} E_{0} & \to E_{0}+\alpha_{E}\cos\left(\omega_{\alpha }t\right) & & \\ I_{0} & \to I_{0}+\alpha_{I}\cos\left(\omega_{\alpha }t+\phi_{\alpha }\right) & \end{array}$$

Here the amplitudes (α*_E_*, α*_I_*) of the background modulations are constrained to be less than the constant background (*E*_0_, *I*_0_). We introduce a phase offset ϕ_α_ to allow for differences in local processing of the modulatory input by the excitatory and inhibitory subpopulations, as might occur if they have different membrane time constants.

### Simulations and data analysis

All of the simulations carried out here were performed using the built in ode solver in MATLAB (Natick, MA, USA), *ode23*. We considered several types of inputs *P*(*t*) – step function, impulse, sinusoidal, and random noise and found the essential characteristics of the system response were represented by the step function input. The spectrum of the model output was analyzed using a FFT in MATLAB (Mathworks, Natick, MA, USA). For sustained oscillations in the limit cycle regime, we also analyzed the model outputs either by using Hilbert Transforms to estimate the frequency and amplitude of the oscillation or by a complex Morlet wavelet transform. For the damped oscillations, we fit the oscillation to a damped sinusoid exp(*j*2 π*ft*(1 + *j*γ)) where *f* is the frequency of the oscillation and γ is the damping coefficient. We obtained direct estimates of frequency using zero crossings and estimated the damping coefficient by fitting an exponential to the decay of amplitude across cycles of the oscillation.

## Results

### Basic response properties of WC oscillator

**Figure 2:**
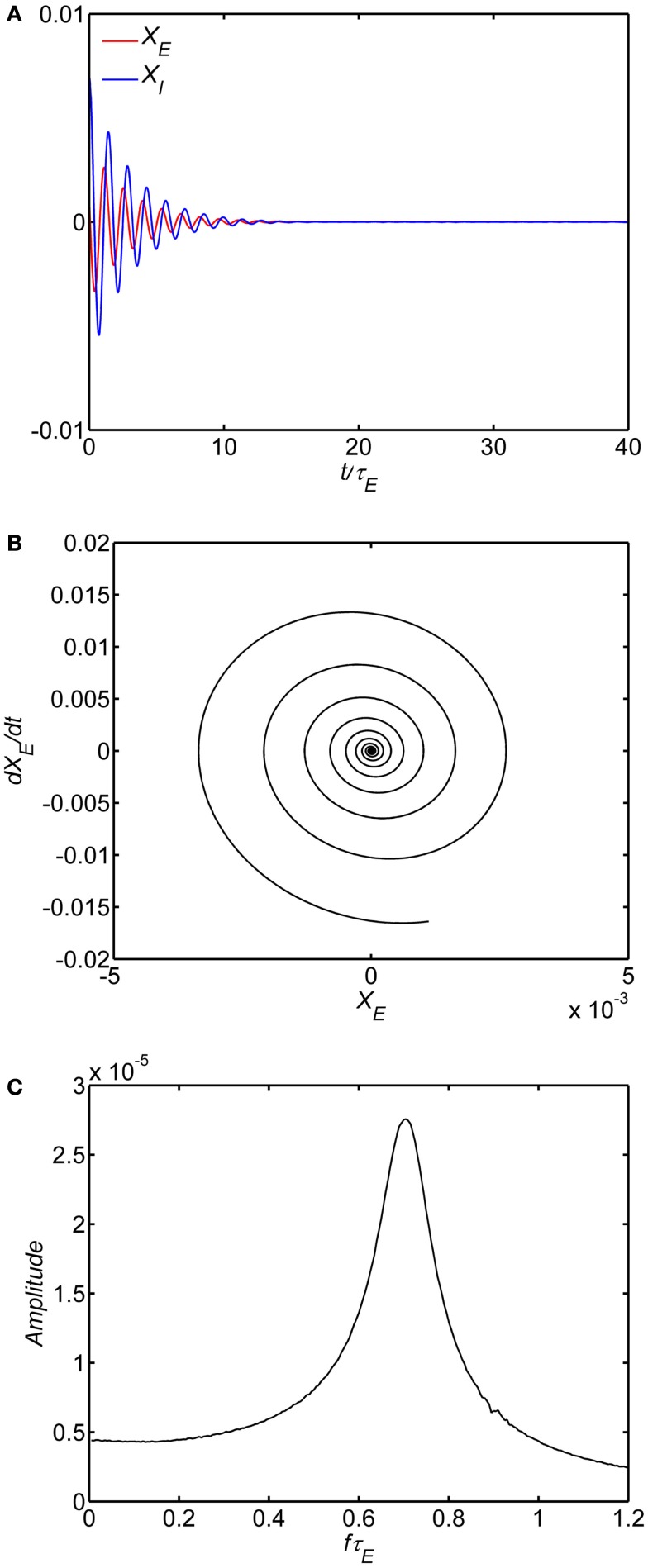
**Damped oscillation regime of the model**. In all of the simulations, the following parameters are fixed: (1) The ratio of time constants A = τ_*I*_/τ_*E*_ = 1, (2) the background state *E*_0_(*t*) = *I*_0_(*t*) = 0.25, (3) the connection weights are (*w_EI_*, *w_IE_*, *w_II_*) = (50,15,0) and (4) the input *P*(*t*) = 0.1 is a step function at time 0. Damped oscillation observed with self-excitation *w_EE_* = 12. The time series of the excitatory and inhibitory subpopulations are shown in **(A)**. In these plots time is normalized by excitatory membrane time constant τ_*E*_. Phase-plane plots for the excitatory. subpopulation are shown in **(B)**. Amplitude spectra obtained by the FFT are shown in **(C)**. Normalized frequency is *f*τ_*E*_. If τ_*E*_ = 20 ms, a normalized frequency of 1 corresponds to 50 Hz.

**Figure 3:**
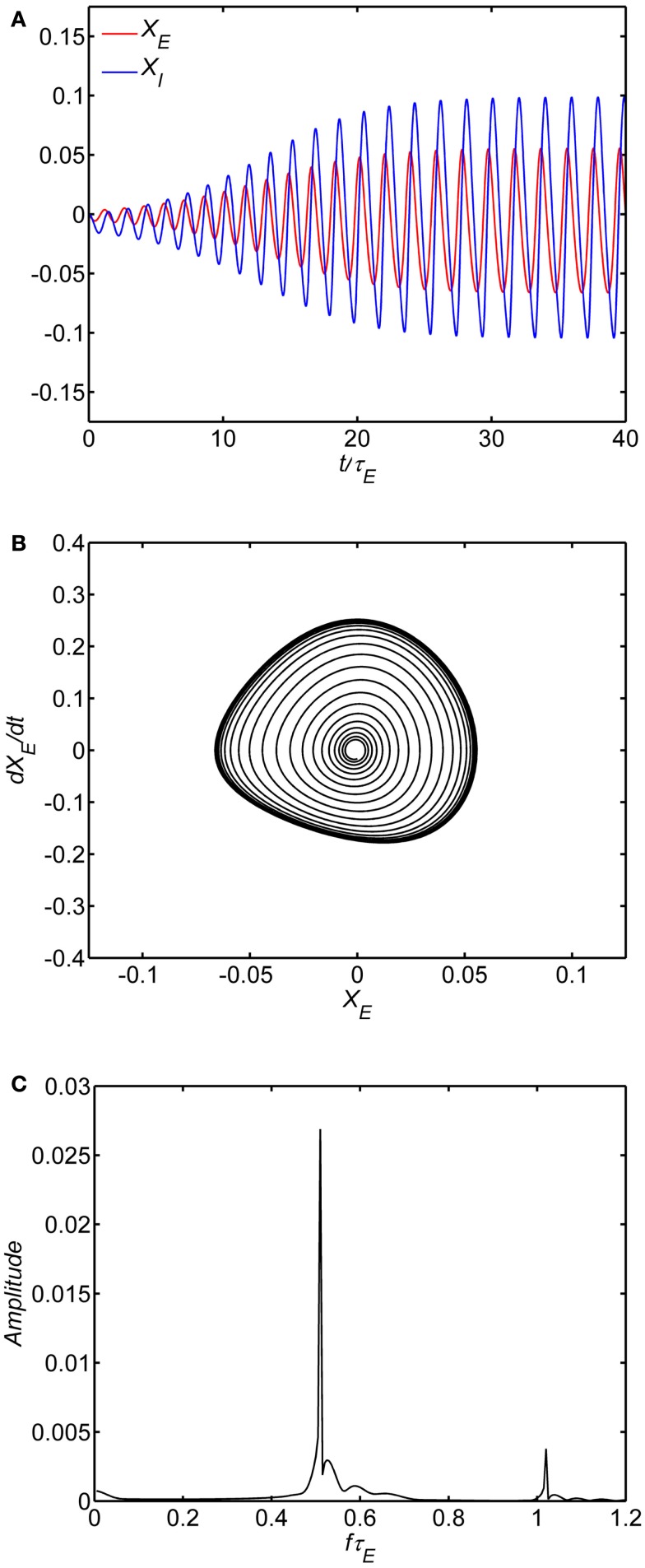
**Limit cycle regime of the model**. In all of the simulations, the following parameters are fixed: (1) The ratio of time constants *A* = τ_*I*_/τ_*E*_ = 1, (2) the background state *E*_0_(*t*) = *I*_0_(*t*) = 0.25, (3) the connection weights are (*w_EI_*, *w_IE_*, *w_II_*) = (50,15,0) and (4) the input *P*(*t*) = 0.1 is a step function at time 0. Limit cycle oscillation observed with self-excitation *w_EE_* = 20. The time series of the excitatory and inhibitory subpopulations are shown in **(A)**. In these plots time is normalized by excitatory membrane time constant τ_*E*_. Phase-plane plots for the excitatory subpopulation are shown in **(B)**. Amplitude spectra obtained by the FFT are shown in **(C)**. Normalized frequency is *f*τ_*E*_. If τ_*E*_ = 20 ms, a normalized frequency of 1 corresponds to 50 Hz.

We first examined the behavior of the system with identical excitatory and inhibitory time constants (*A* = τ_*1*_/τ_*E*_ = 1) and a fixed background state (*E*_0_ = *I*_0_ = 0.25). The specific value of *w_EE_* separating limit cycle from damped oscillations depends on the background excitability as in Eq. 8; with *E*_0_ = *I*_0_ = 0.25 the critical value is *w_EE_* = 15. The limit cycle is observed if the self-excitation is sufficiently large (*w_EE_* > 15); smaller values lead to damped oscillations of higher frequency. An example of numerical solutions for the model with the self-excitation (*w_EE_*) parameter in the damped oscillation range is shown in Figure 2. In the time series plot (Figure 2A), the time variable is normalized with respect to the excitatory membrane time constant τ_*E*_ and in the amplitude spectra (Figure 2C) the frequency variable *f* is normalized as *f* τ_*E*_ For example, if τ_*E*_ falls in the range of 10–20 ms range, the damped oscillation corresponds to a gamma band oscillation in the 35–70 Hz band. The limit cycle is observed if the self-excitation is sufficiently large (Figure 3). The limit cycle has a lower fundamental frequency as shown in the spectrum in Figure 3C; if τ_*E*_ falls in the range of 10–20 ms range, the dominant frequency is in the 25–50 Hz range and also exhibits harmonics (Second harmonic shown).

**Figure 4:**
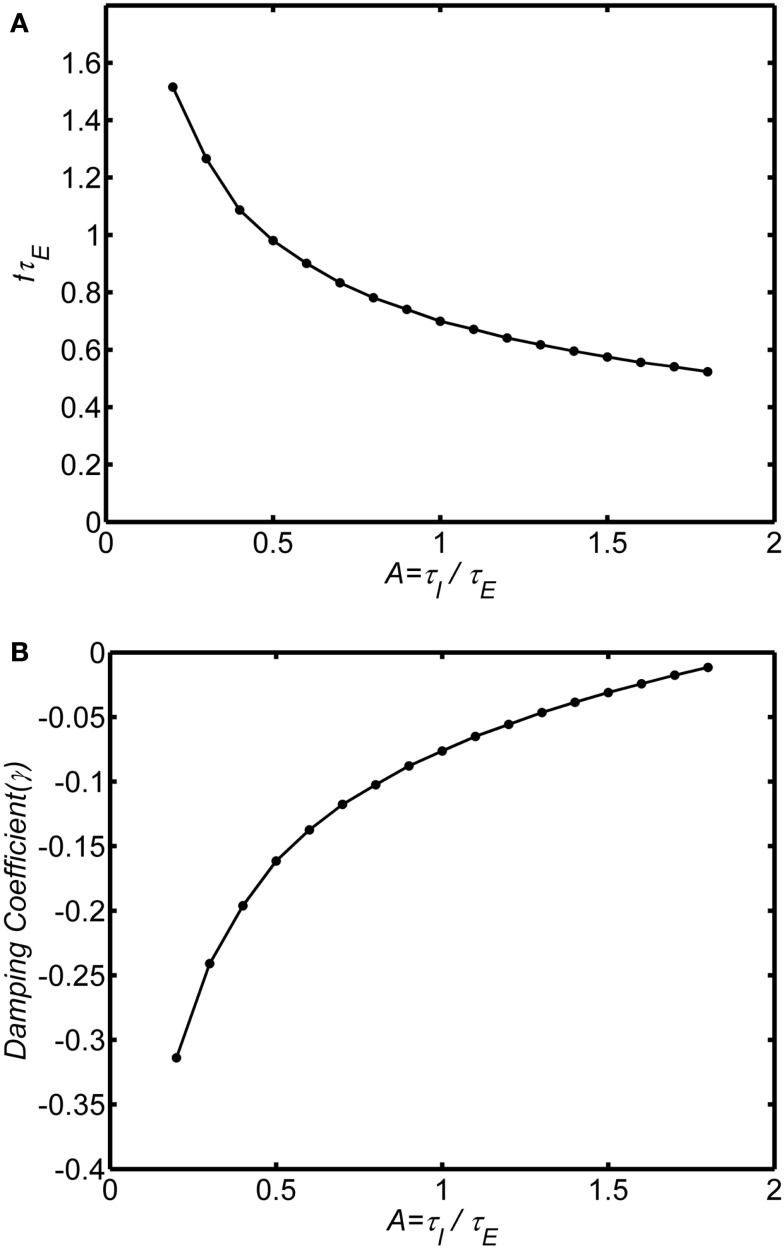
**Effect of the ratio of inhibitory to excitatory time constants (*A* = τ_*I*_/τ_*E*_) on the damped oscillations**. System connection weights are fixed as (*w_EI_*, *w_IE_*, *w_II_*, *w_EE_*) = (50,15,0, 12). The background state is fixed as *E*_0_(*t*) = *I*_0_(*t*) = 0.25. Input is a step function of magnitude *P*(*t*) = 0.1. Frequency was estimated by analyzing zero crossings. Damping was estimated by fitting an exponential decay to the peaks of a rectified (absolute value) of the time series. **(A)** Normalized frequency is *f*τ_*E*_. If τ_*E*_ = 10 ms, a normalized frequency of 1 corresponds to 100 Hz. **(B)** Damping coefficient.

The ratio of inhibitory to excitatory time constants *A* influences both the frequency and damping of the oscillations. Figures 4A,B show the oscillation frequency and damping coefficient in the damped oscillation regime for self-excitation in the damped oscillation range (*w_EE_* = 12). When the inhibitory time constant is smaller than the excitatory time constant (*A* < 1) the oscillations are highly damped, but if the inhibitory time is constant is larger than the excitatory time constant (*A* > 1) the oscillations are weakly damped. Thus, in order to observe the damped oscillations, it must be the case that inhibitory time constants are longer than the excitatory time constant. As the ratio *A* increases further the system will eventually transition to a limit cycle oscillation.

**Figure 5:**
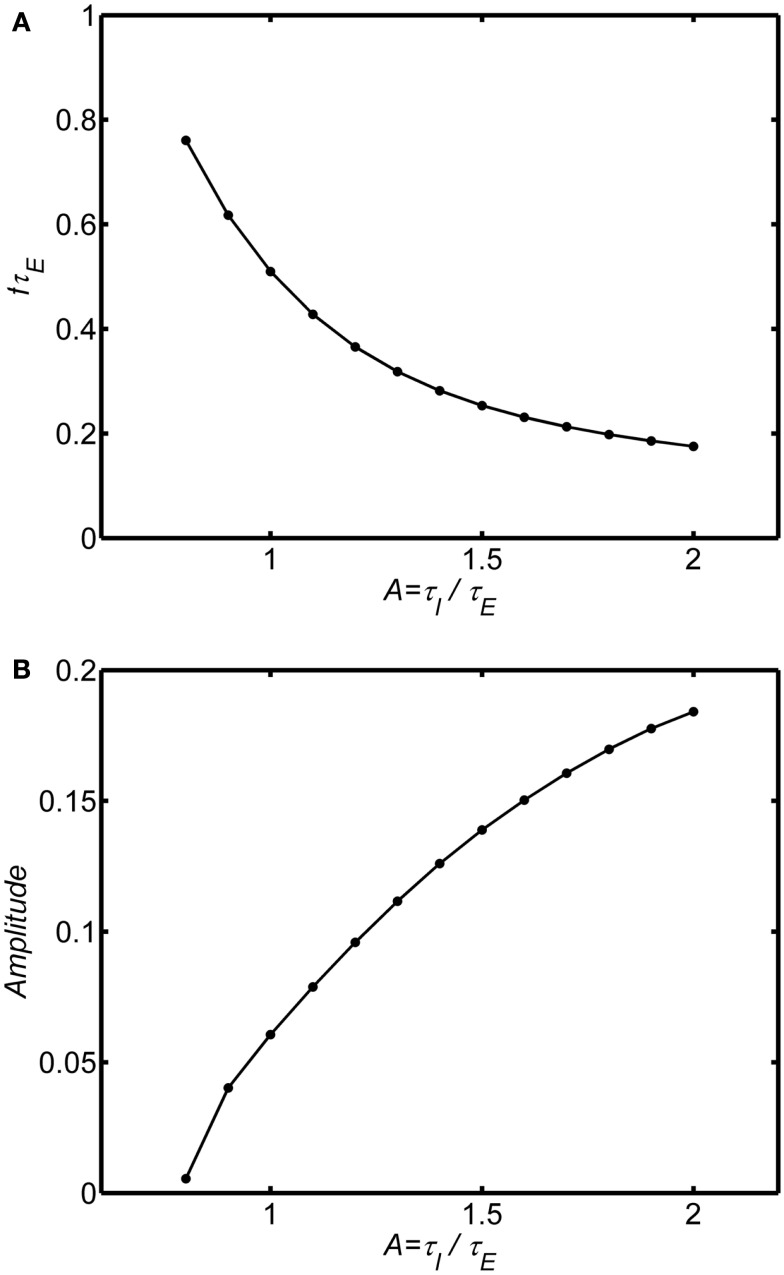
**Effect of the ratio of inhibitory to excitatory time constants in the limit cycle regime**. The background state is fixed as *E*_0_(*t*) = *I*_0_(*t*) = 0.25. Input is a step function of magnitude *P*(*t*) = 0.1. System connection weights are fixed as (*w_EI_*, *w_IE_*, *w_II_*) = (50,15,0) and *w_EE_* = 18 to fix the system in the limit cycle regime. Frequency and amplitude estimated using a Hilbert Transform of the period from of 100τ_*E*_ to 200τ_*E*_. Normalized frequency is *f*τ_*E*_. If τ_*E*_ = 10 ms, normalized frequency of 1 is 100 Hz. For *A* smaller than the range shown for each plot the limit cycle transitions to a damped oscillation. The main result is that increasing τ_*E*_ relative to τ_*I*_ reduces damping (increasing amplitude) and lowers the frequency of the oscillation. **(A)** Normalized frequency **(B)** Amplitude.

Figures 5A,B shows an example with the self-excitation parameter in the limit cycle regime (*w_EE_* = 18). As *A* increases the frequency of the oscillation decreases and the amplitude increases consistent with reduced damping in the system. Essentially, for any level of self-excitation *w_EE_*, as inhibitory time constant increases, damping is reduced, and frequency decreases. The frequency range of the limit cycle oscillations is much lower than the damped oscillation. For τ_*E*_ = 10 ms the damped oscillations are in the high gamma band frequency range (60–100 Hz) when *A* ranges from 0.5–1.5. In contrast, for the same range of τ_*E*_ and *A* ranging from 1–2 the limit cycle oscillations range is in the lower gamma frequency range (20–50 Hz).

### Effect of background state

**Figure 6:**
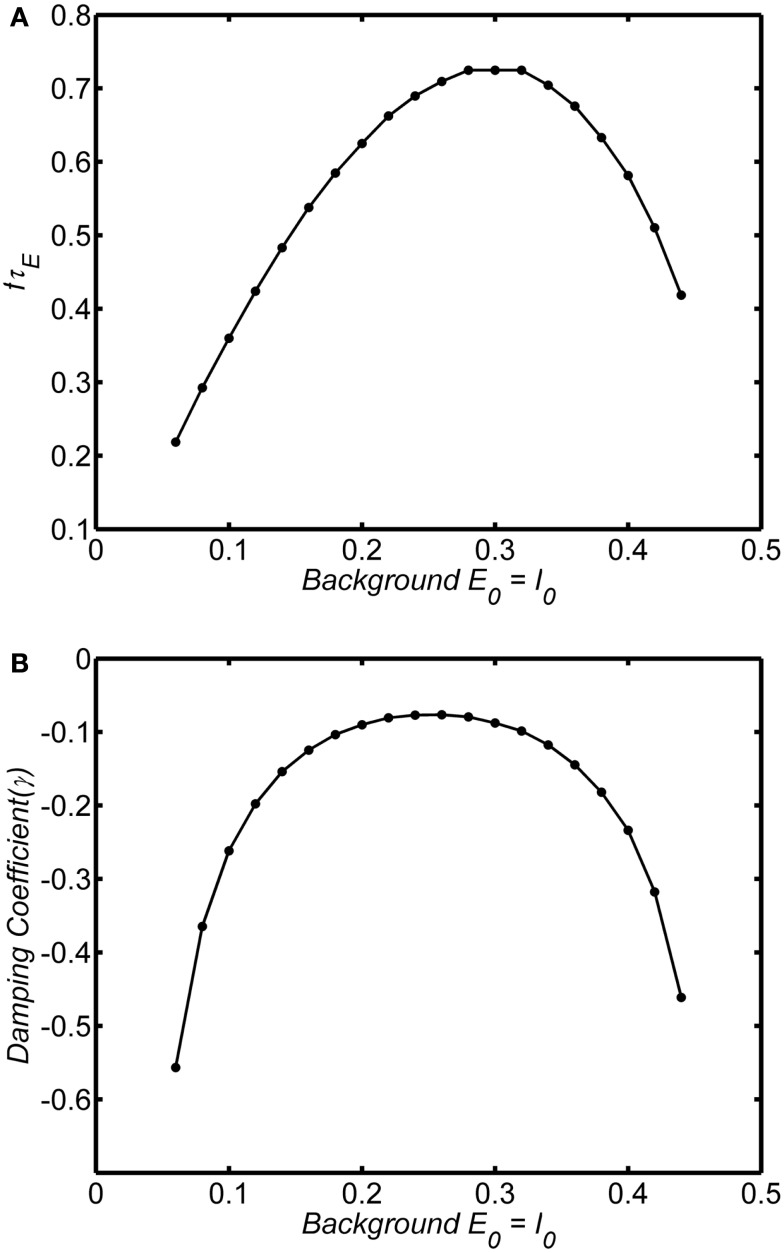
**Dependence of frequency and damping on background state for the damped oscillations**. System connection weights are fixed as (*w_EI_*, *w_IE_*, *w_II_*, *w_EE_*) = (50,15,0, 15). Input is a step function of magnitude 0.1. We have verified that the curves are the same for step functions up to 0.3 Frequency was estimated by analyzing zero crossings. Damping was estimated by fitting an exponential decay to the peaks of a rectified (absolute value) of the time series. Normalized frequency is *f*τ_*E*_. If τ_*E*_ = 20 ms, normalized frequency of 1 corresponds to a 50 Hz oscillation. **(A)** Normalized frequency **(B)** Damping coefficient.

The frequency and damping of the WC oscillator is strongly influenced by the background state. Figures 6A,B shows the frequency and damping as a function of background state variables *E*_0_ = *I*_0_ for an example in the damped oscillation regime (*w_EE_* = 12; *A* = 1). At very low levels of background activity the system exhibits low frequency rapidly damped oscillations. As the background activity increases above *E*_0_ = *I*_0_ = 0.1 the oscillations become weakly damped and frequency increases as damping decreases. Damping reaches a minimum at *E*_0_ = *I*_0_ = 0.25 and the frequency of the oscillation reaches a peak at *E*_0_ = *I*_0_ = 0.3. At higher levels of background activity, the oscillations decrease in frequency and are again highly damped. Thus only at the center of the range, at around 0.2–0.3 can we observe high frequency weakly damped oscillations.

**Figure 7:**
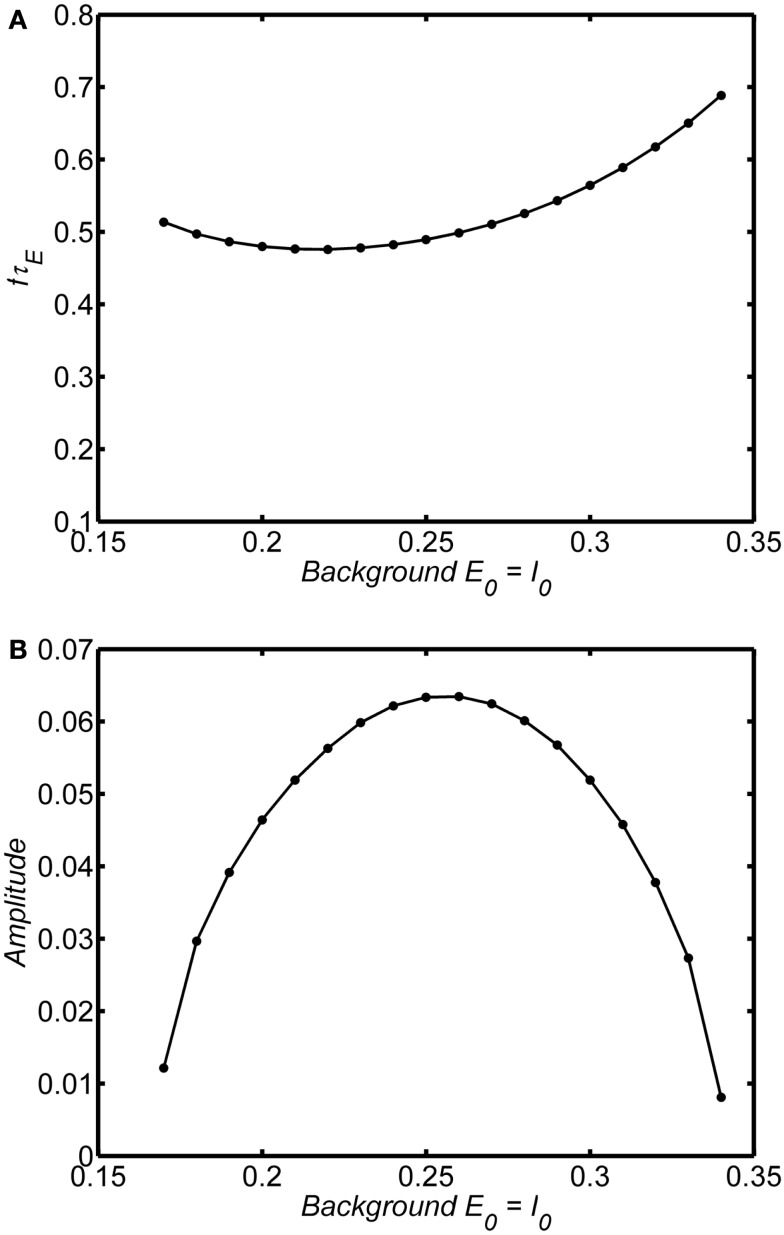
**Dependence of frequency and amplitude of limit cycle oscillations on the background state**. System connection weights are fixed as (*w_EI_*, *w_IE_*, *w_II_*, *w_EE_*) = (50,15,0, 20) (dashed line) or (*w_EI_*, *w_IE_*, *w_II_*, *w_EE_*) = (50,15,0, 25) (solid line). Input is a step function of magnitude 0.1. Frequency and amplitude estimated using a Hilbert Transform of the period from of 100τ_*E*–_200τ_*E*._ Normalized frequency is *f*τ_*E*._ If τ_*E*_ = 20 ms, normalized frequency of 1 corresponds to a 50 Hz oscillation. Outside the range of background states shown there are no limit cycle oscillation for each level of *w_EE_* and the oscillations damp out. The main point is that the amplitude of the limit cycle oscillations depend strongly on the background state. Compared to the damped oscillations (Figure 6) the limit cycle frequency does not depend strongly on the background state. **(A)** Normalized frequency **(B)** Amplitude.

If the system is in the limit cycle regime the same essential damping behavior is observed as shown for an example in Figures 7A,B (*w_EE_* = 18; *A* = 1). The limit cycle amplitude is suppressed above and below *E*_0_ = *I*_0_ = 0.25; at very large or very small values of the background state the limit cycle disappears and is replaced by a damped oscillation. The range of background activity over which the limit cycle is observed can be expanded by increasing the self-excitation parameter (*w_EE_*). However, in contrast to the damped oscillation regime, in the limit cycle the frequency of the oscillations is much less dependent on the background activity level, remaining stable over the range of background states with the high amplitude.

### Effects of top-down signaling

It is increasingly appreciated that neural populations are subject to top-down signals reflected in oscillations in large-scale cortical networks. We modified the WC system equations to incorporate dynamic modulation of background state as in Eq. A12. For simplicity we modulated the background state variables (*E*_0_, *I*_0_) with a sinusoidal signal of fixed frequency; these modulatory frequencies are much slower than the intrinsic frequencies of the WC oscillator. The presence of the modulatory signal alone was not sufficient to drive the system – excitatory input *P*(*t*) was always required.

**Figure 8:**
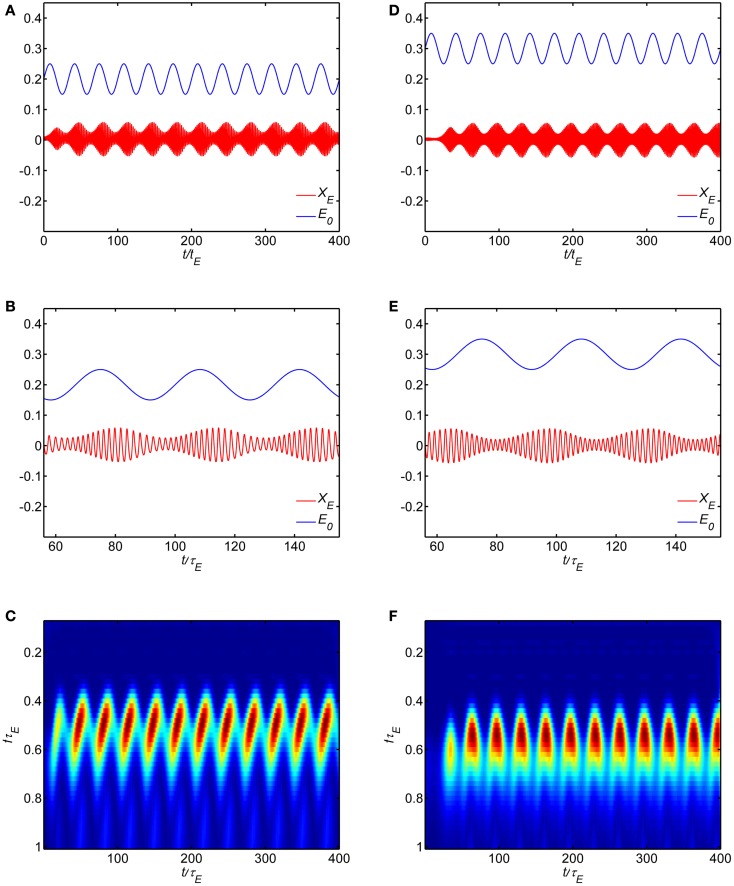
**Dynamic modulation of background state produces cross-frequency coupling in the model**. Model parameters are in the limit cycle regime (*w_EI_*, *w_IE_*, *w_II_*, *w_EE_*) = (50,15,0, 18). (**A–C**) correspond to a background state *E*_0_ = *I*_0_ = 0.2 while **(D–F)** correspond to a background state *E*_0_ = *I*_0_ = 0.3. **(A,B,D,E)** show the time series while **(C,F)** show the wavelet spectrum.

Figure 8 shows some example simulations of the model with the self-excitation parameter set in the limit cycle regime (*w_EE_* = 18). The main effect of the dynamic modulation of background state is to modulate the amplitude and frequency of the oscillation. Figures 8A,B show the time course of a modulatory signal and the oscillation in the WC model. In this example, the modulatory signal is an oscillation about a background state *E*_0_ = *I*_0_ = 0.2 with normalized frequency *f*τ_*E*_ = 0.03. The oscillation in population activity can be seen to modulate in amplitude at the rate of the modulatory signal, with higher amplitude when background activity increases. Thus the phase of the modulatory signal modulates the amplitude of the oscillation. Figures 8D,E show another example where the modulatory signal is an oscillation about a background state *E*_0_ = *I*_0_ = 0.3. Here a different phase relationship is evident with higher amplitude when the background activity decreases. For each example, the temporal evolution of the spectrum obtained with wavelet transform is shown in Figures 8C,F. In these examples, the population oscillates at roughly *f*τ_*E*_ = 0.5 with amplitude modulated at *f*τ_*E*_ = 0.03. If τ_*E*_ = 10 ms, the underlying oscillation frequency is in the gamma band at approximately 50 Hz and the modulation is in the theta band at 3 Hz. In both cases, during each cycle of the modulatory signal as the amplitude of the population activity increases the frequency decreases.

We carried out simulations over a broad range of parameters to determine if we could produce the apparent effect of amplitude modulation of the intrinsic limit cycle oscillation by adding a sinusoidal modulation at low frequencies to the input *P*(*t*). In no case were we able to reproduce the amplitude modulation shown in Figure 8, and the limit cycle show stable amplitude and frequency.

## Discussion

In this paper we have revised the Wilson–Cowan model of the interactions within a population of excitatory and inhibitory neurons in order to investigate the impact of background activity on the dynamics of neural populations. In our model this background state is determined statically at very long time scales (presumably by neuromodulator systems) and dynamically at faster time scales by the activity of other cortical systems that exert top-down control on the neural population. We find that our model formalizes the mechanisms by which background state can influence local population dynamics consistent with observations in experimental studies in different behavioral contexts and recording methods.

### Dynamics of the Wilson–Cowan system

The basic response properties of the system around a fixed background state indicate that the system produced damped oscillations or sustained limit cycle oscillations depending on the level of self-excitation of the excitatory neurons and the relative value of excitatory and inhibitory time constants. Linear analysis about critical (equilibrium, fixed) points indicates that for lower values of self-excitation damped oscillations will be observed while at higher values of self-excitation limit cycle oscillations are observed. In addition, sustained oscillations are more likely to be observed within local populations with longer inhibitory time constants than excitatory time constants. When excitatory time constants are longer than the inhibitory time constants, rapidly damped high frequency oscillations are observed in the system. Inhibitory time constants that are longer than the excitatory time constants in the 10 ms range result in higher amplitude lower frequency oscillations in the gamma band (30–100 Hz) and generally support limit cycle oscillations rather than damped oscillations. In cases where the inhibitory time constants are much longer these sustained oscillations can be produced at even lower frequencies in the beta (13–30 Hz) and alpha (8–12 Hz) ranges.

Whereas early studies of membrane time constants in mammalian cortex were very short, typically less than 10 ms, more modern studies with improved recording methods report the wide range up to 100 ms (Koch et al., 1996). In particular, very long inhibitory time constants have been reported for thalamic and cortical populations. Thus, we can reasonably expect the ratio of inhibitory to excitatory time constants to be significantly larger than one, supporting the existence of linear instability and corresponding limit cycle oscillations over a broad range of model parameters. Such self-sustained dynamics may contribute locally to the generation of spontaneous EEG rhythms.

### Effects of background state on population dynamics

The dynamics of the model population depend very strongly on the background state in both limit cycle and damped oscillation ranges of the parameters. At very low levels of background activity (*E*_0_, *I*_0_ < 0.2), the population does not respond to the external input. As background activity increases the system responds to external input. In the limit cycle regime, the *amplitude* of the oscillation depends strongly on the background activity while in the damped oscillation regime the *frequency* of the oscillations depends strongly on the background activity.

The overall level of background activity is determined by the neuromodulatory systems that control the sleep-wake cycles, level of arousal, and the response to reward and/or threat. Although there are variations in the densities of neuromodulator inputs to different cortical areas, almost all cortical areas receive neuromodulatory input (Goldman-Rakic et al., 1990). Changes in these states occur over very long time scales; in an awake, behaving animal (or human) these tonic influences are generally considered constant. Thus in general, we can expect that the background level is constant over a responsive level of the population, although there are also phasic modulations of the cholinergic and dopaminergic neuromodulator systems that may play a role in stimulus response and reward seeking behavior (Sarter and Bruno, 1997; Chuhma et al., 2004; Zhang and Sulzer, 2004), and can be expected to have dynamic influences on local networks.

### Modulation of population dynamics by top-down signals

The dynamic modulation of background state creates amplitude and frequency modulation of the intrinsic gamma oscillations of the WC system. The most salient effect is amplitude modulation by the phase of slow modulations of the background states. In the experimental literature this phenomenon is explained as dynamic modulation of the excitability of the population (Jensen and Colgin, 2007). Our model captures this essential behavior, and provides a plausible mechanism to incorporate these types of effects in computational models. In our model, the specific phase/amplitude relationship was influenced by the overall activity level; the specific phase of the modulation that produced robust oscillations was arbitrary. The experimental literature is consistent with this picture, with different studies reporting different phases of the modulation signal for peak amplitude of the local oscillation as shown in Figure 1 of (Lisman and Buzsaki, 2008).

This essential phenomenon of cross-frequency coupling has been observed in animal models in a number of experimental contexts (Buzsaki and Draguhn, 2004; Scheffzuk et al., 2011) and human ECoG recordings (Canolty and Knight, 2010; Voytek et al., 2010). These findings have since been confirmed in human EEG where the phase of the theta rhythm is shown to modulate the amplitude of the gamma rhythm (Demiralp et al., 2007). It has long been known that the phase of the alpha rhythm at stimulus onset influences amplitude and phase of the evoked potential (Dustman and Beck, 1965; Jansen and Brandt, 1991; Gruber et al., 2005; Hanslmayr et al., 2007). Moreover, there are a number of studies that have shown that the state of the cortex (as measured by EEG oscillations) can predict the perception of a sensory stimulus, presumably by modulating the sensory evoked response (Haig and Gordon, 1998; Hanslmayr et al., 2007).

### Local-global interactions

We have proposed a conceptual framework in which local networks (cell assemblies) are embedded in a global environment that produces standing waves due to propagation in the corticocortical (white matter) fibers and periodic boundary conditions (Nunez, 1995, 2000; Nunez and Srinivasan, 2006). That is, the neocortex and underlying white matter are modeled as a closed loop or spherical shell. In this paper, we have a proposed a method to model the top-down influences of such systems on a local network. These top-down influences may be the result of feedback from global network. In our analysis we have isolated the local network from the global system, and only analyzed the local network dynamics. Similarly, global models typically assume that the local network is sufficiently localized such that its (bottom up) influence on the global dynamics may be neglected to first approximation. This condition might be satisfied in the eyes closed resting state, for example. On the other hand, the (eyes open) processing of substantial visual input or complex cognitive functions may involve multiple local thalamocortical networks that act (bottom up) to modify the global networks that are influencing the local networks. Future work must explicitly consider in more detail how the local networks and global networks interact.

Our study suggests that models of these local networks must incorporate the idea that the response properties of the networks can be modified by modulatory inputs. In our modified WC model, the addition of oscillatory afferent input does not modify the system dynamics. We explicitly incorporated dynamic modulation of the system properties by making the background state an explicit part of the model. In most models (including the original WC) the background state of the neurons is mathematically removed, and the dynamics of the system is studied without further consideration of the background state. This approximation is limiting; modification of the background state may be an important mechanism of top-down signaling in the cortex, especially in the control of goal-oriented behavior such as attention. Local networks in the cortex experience dynamic background states which can be readily incorporated into most model formulations. This may also have importance in specific models that seek to make a distinction between feedforward and feedback connections in sensory systems (Mountcastle, 1997; Lamme and Roelfsema, 2000).

## Conclusions

Since the first human recording in the early 1920s the physiological bases for the wide range of rhythmic EEG activity has been somewhat of a mystery. As one important “window on the mind,” EEG has long provided a critical tool in pursuit of connecting neural dynamics to cognitive processes. Human brains produce a proverbial “spectral zoo” that is closely correlated to behavior and cognition. A major obstacle in this quest is a shortage of robust and widely appreciated theoretical support for EEG’s dynamic behavior in time and spatial location over the scalp. The conceptual framework facilitated by such theory could have a substantial influence on the design of new EEG-cognitive experiments. In this paper, we propose an approach to incorporate global (top-down) influences on local networks. The essence of our approach is to immerse the local network in a dynamic background state. These dynamics could be generated by a global model of interactions across the cortex; they could also be modeled from experimental EEG data. This approach is sufficiently general to be applied to other theoretical formulations of population dynamics in neural populations and to models of specific cognitive influences on local circuit dynamics.

## Conflict of Interest Statement

The authors declare that the research was conducted in the absence of any commercial or financial relationships that could be construed as a potential conflict of interest.

## Appendix

### The basic WC analysis

The classical WC model (Wilson and Cowan, 1972, 1973) applies to populations of interacting excitatory and inhibitory neurons in some local neocortical region as indicated in Figure 1. An important WC assumption is that axon propagation delays are negligible; that is, all WC delays are due to PSP rise and decay times. WC is then a strictly local model and represents the opposite limiting case to global models in which delays are axonal, especially in the longer corticocortical axons forming most of human white matter (Nunez, 1974; Nunez and Srinivasan, 2006). These distinct local and global models have been shown to be fully compatible and may be combined into local/global models (Jirsa and Haken, 1996; Nunez, 2000).

The basic WC dependent variables are the *fractions of excitatory and inhibitory active cells* (dimensionless action potential densities) *E*(*t*), *I*(*t*), which can evidently exhibit very high frequency jitter not treated in the analysis. Rather, the WC equations are expressed in terms of coarse grained (in time) excitatory 〈*E*(*t*)〉 and inhibitory 〈*I*(*t*)〉 action potential densities, where the critical point 〈*E*(*t*)〉, 〈*I*(*t*)〉 = (0,0) is considered by WC to be an equilibrium background state occurring when the external (afferent) driving function *P*(*t*) = 0. Thus, the variables 〈*E*(*t*)〉, 〈*I*(*t*)〉 are allowed to take on negative values by WC, an inaccurate and (as we show here) unnecessary approximation to their physiological interpretation as fractions of active cells. The WC equations (1.3.1 and 1.3.2 from the 1973 paper) are

(A1) $$\begin{array}{rcll} \tau_{E}\frac{d\left\langle E\left(t\right)\right\rangle }{dt} & =-\left\langle E\left(t\right)\right\rangle +\left[1-r_{E}\left\langle E\left(t\right)\right\rangle \right]S_{E}\left[P\left(t\right)\right. & & \\ & \left.\;+w_{EE}\left\langle E\left(t\right)\right\rangle -w_{IE}I\left(t\right)\right] & & \\ \tau_{I}\frac{d\left\langle I\left(t\right)\right\rangle }{dt} & =-\left\langle I\left(t\right)\right\rangle +\left[1-r_{I}\left\langle I\left(t\right)\right\rangle \right]S_{I}\left[w_{EI}\left\langle E\left(t\right)\right\rangle \right. & & \\ & \left.\;-w_{II}\left\langle I\left(t\right)\right\rangle \right] & \end{array}$$

Here we drop the spatial dependence *x* of all variables since axon speeds are assumed to be infinite implying that neural spatial separations have no effect on dynamic behaviors in this approximation. We also distinguish between the excitatory and inhibitory membrane time constants τ_*E*_, τ_*I*_. In addition, we only allow excitatory afferent input *P*(*t*).

### Some issues with the original WC analysis

1. WC write *the proportion of sensitive excitatory cells* (neurons currently firing or in their refractory periods *r_E_*) as $R_{E}\left(t\right)=1-\overset{t}{\underset{t-r_{E}}{\int }}E\left(t^{\prime }\right)dt^{\prime }$. This is not dimensionally correct and leads to the dimensionally incorrect Eq. A1. The WC equation also yields the incorrect result *R_E_*(*t*) → 1 as the refractory period *r_E_* → 0. The correct expression is
  (A2) $$\begin{array}{rcll} & R_{E}\left(t\right)=1-\frac{1}{r_{E}}\overset{t}{\underset{t-r_{E}}{\int }}E\left(t^{\prime }\right)dt^{\prime } & & \\ & R_{E}\left(t\right)\to 1-E\left(t\right)\text{ when }r_{E}\to 0 & \end{array}$$
  In this limit, the sensitive population *R*_E_(*t*) consists of all cells not firing at time *t*. The excitatory and inhibitory integrals should have been divided by the refractory times *r_E_*, *r_I_*, equivalent to setting *r_E_*, *r_I_* = 1, in Eq. A1, a simple corrective step often adopted by others using the WC model.
2. Negative values of 〈*E*(*t*)〉, 〈*I*(*t*)〉 are not realistic physiologically for essentially the same reason. During times when *E*(*t*), *I*(*t*) < 0, the fractions of sensitive cells *R_E_*(*t*), *R_I_*(*t*) > 1, which is also inconsistent with realistic physiology. By contrast, the modified WC model presented here forces
  $$\left\langle E\left.\left(t\right)\right\rangle \right.,\left\langle I\left.\left(t\right)\right\rangle \geq 0,\;\text{for}\;-\infty \right.<t<+\infty .$$
3. WC choose sigmoid response functions such that *S_E_*(0) = *S_I_*(0) = 0 in order to force (0,0) to be a critical (equilibrium) point where the variable time derivatives equal zero. Such critical points may be either state or unstable. If a critical point is stable, any brain dynamic state coming sufficiently close to this point will become fixed (forever static or “brain dead”). Of more interest to us are unstable critical points associated with on-going oscillations (limit cycles), possibly underlying EEG. In WC, the tissue response functions can become negative, a physiological impossibility; thus, in our following modified analysis, the WC conditions are replaced by
  (A3) $$\begin{array}{rcll} S_{E}\left[P\left(t\right),\left\langle E\left(t\right)\right\rangle ,\left\langle I\left(t\right)\right\rangle \right]\geq 0 & & & \\ S_{I}\left[\left\langle E\left(t\right)\right\rangle ,\left\langle I\left(t\right)\right\rangle \right]\geq 0 & & \end{array}$$
4. WC interpret the WC parameter μ as a single membrane time constant. Based on the classic solution of the cable equation and the distribution of excitatory synapses on dendrites with inhibitory synapses typically near cell bodies and our increased appreciation of wide ranges of excitatory and inhibitory time constants τ_*E*,_ τ_*I*_, we consider casesτ_*E*_ ≠ τ_*I*_.

### A modified version of the WC analysis

Define the non-dimensional time $t_{1}=\frac{t}{\tau_{E}}$ and time constant ratio $A=\frac{\tau_{I}}{\tau_{E}}$ so that with rescaled variables Eq. A1 become

(A4) $$\begin{array}{rcll} \frac{d\left\langle E\right\rangle }{dt_{1}} & =-\left\langle E\right\rangle +\left(1-\left\langle E\right\rangle \right)S_{E}\left(P+w_{EE}\left\langle E\right\rangle -w_{IE}\left\langle I\right\rangle \right) & & \\ A\frac{d\left\langle I\right\rangle }{dt_{1}} & =-\left\langle I\right\rangle +\left(1-\left\langle I\right\rangle \right)S_{I}\left(w_{EI}\left\langle E\right\rangle -w_{II}\left\langle I\right\rangle \right) & \end{array}$$

Here the non-dimensional parameter *A* may range from somewhat less than one to as high as perhaps 5. For convenience we drop the subscript 1 on the non-dimensional time variable. We introduce the new dependent variables *X_E_*(*t*), *X_I_*(*t*), which provide perturbations about the critical point (*E*_0_, *I*_0_)

(A5) $$\begin{array}{rcll} \left\langle E\left(t\right)\right\rangle & =E_{0}+X_{E}\left(t\right) & & \\ \left\langle I\left(t\right)\right\rangle & =I_{0}+X_{I}\left(t\right) & \end{array}$$

We assume that the input function *P*(*t*) is exclusively excitatory such that *P*(*t*) ≥ 0 at all times. The following conditions follow from the variable definitions

(A6) $$\begin{array}{rcll} & 0\leq E_{0}+X_{E}\left(t\right)\leq 1 & & \\ & 0\leq I_{0}+X_{I}\left(t\right)\leq 1 & \end{array}$$

Equations (A4) then yield

(A7) $$\begin{array}{rcll} \frac{dX_{E}}{dt}= & -E_{0}-X_{E}+\left(1-E_{0}-X_{E}\right)S_{E}\left[P+w_{EE}\left(E_{0}+X_{E}\right)\right. & & \\ & \left.-w_{IE}\left(I_{0}+X_{I}\right)\right] & & \\ A\frac{dX_{I}}{dt}= & -I_{0}-X_{I}+\left(1-I_{0}-X_{I}\right)S_{I}\left[w_{EI}\left(E_{0}+X_{E}\right)\right. & & \\ & \left.-w_{II}\left(I_{0}+X_{I}\right)\right] & \end{array}$$

The sigmoid functions *S_E_*, *S_I_* are chosen here such that (*E*_0_, *I*_0_) is a critical point when *P*(*t*) = 0. Thus, we choose the following alternate sigmoid tissue response functions.

(A8) $$\begin{array}{rcl} S_{I} & =\frac{1}{1+\exp\left[-w_{EI}\left(E_{0}+X_{E}\right)+w_{II}\left(I_{0}+X_{I}\right)+K_{I}\right]} & \end{array}$$

(A9) $$\begin{array}{rcl} S_{E} & =\frac{1}{1+\exp\left[-w_{EE}\left(E_{0}+X_{E}\right)+w_{ie}\left(I_{0}+X_{I}\right)+K_{E}\right]} & \end{array}$$

The constants *K_E_*, *K_I_* are added to the original WC sigmoid functions, thereby determining the response range during oscillations about fixed points. This choice of the forms of the sigmoid response functions insures that (1) (*E*_0_, *I*_0_) or (*X,Y*) = (0,0) is a critical point and (2) 0 ≤ *S_E_* ≤ 1 and 0 ≤ *S_I_* ≤ 1. Substitution of Eqs A8 and A9 into Eq. A7 and setting *X_E_*(*t*) = *X_I_*(*t*) = 0 yields the sigmoid constants in terms of the critical point

(A10) $$\begin{array}{rcl} K_{I}=w_{EI}E_{0}-w_{II}I_{0}+Log\left[\frac{1}{I_{0}}-2\right] & & \end{array}$$

(A11) $$\begin{array}{rcl} K_{E}=w_{EE}E_{0}-w_{IE}I_{0}+Log\left[\frac{1}{E_{0}}-2\right] & & \end{array}$$

Substitution of Eqs A10 and A11 into Eqs A8 and A9 yields

(A12) $$\begin{array}{rcll} & S_{I}\left[w_{EI}\left(E_{0}+X_{E}\right)-w_{II}\left(I_{0}+X_{I}\right)\right] & & \\ & \;=\frac{1}{1+\left(\frac{1}{I_{0}}-2\right)\exp\left(-w_{EI}X_{E}+w_{II}X_{I}\right)}\;I_{0}<\frac{1}{2} & \end{array}$$

(A13) $$\begin{array}{rcll} & S_{E}\left[w_{EE}\left(E_{0}+X_{E}\right)-w_{IE}\left(I_{0}+X_{I}\right)\right] & & \\ & \;=\frac{1}{1+\left(\frac{1}{E_{0}}-2\right)\exp\left(-w_{EE}X_{E}+w_{IE}X_{I}\right)}\;E_{0}<\frac{1}{2} & \end{array}$$

### Phase-plane analysis

We assume *w_II_* ≅ 0 in all of the following analyses based on our preliminary studies: In simulations with non-zero *w_II_* the effect is only to set the activity level of the system and has no significant influence on the dynamics. The first step in the analysis of Eq. A7 is to find the nature of the critical point (0,0). To accomplish this we expand the functions *F*(*X_E_*, *X_I_*) and *G*(*X_E_*, *X_I_*) about (0,0), where these functions are the expressions on the right sides of Eq. A7, that is

(A14) $$\begin{array}{rcll} F\left(X_{E},X_{I}\right)=-E_{0}-X_{E}+\left(1-E_{0}-X_{E}\right)S_{e} & & & \\ G\left(X_{E},X_{I}\right)=\left[-I_{0}-X_{I}+\left(1-I_{0}-X_{I}\right)S_{i}\right]∕A & & \end{array}$$

Taylor expansion about the critical point (0,0) yields equations of the general form

(A15) $$\begin{array}{rcll} \frac{dX_{E}}{dt} & =F\left(X_{E},X_{I}\right)≅F\left(0,0\right)+{\left(\frac{\partial F}{\partial X_{E}}\right)}_{0}X_{E}+{\left(\frac{\partial F}{\partial X_{I}}\right)}_{0}X_{I} & & \\ & \equiv aX_{E}+bX_{I} & & \\ \frac{dX_{I}}{dt} & =G\left(X_{E},X_{I}\right)≅G\left(0,0\right)+{\left(\frac{\partial G}{\partial X_{E}}\right)}_{0}X_{E}+{\left(\frac{\partial G}{\partial X_{I}}\right)}_{0}X_{I} & & \\ & \equiv cX_{E}+dX_{I} & \end{array}$$

Here we have forced *F*(0,0) = *G*(0,0) = 0 by proper choice of the constants *K_E_*, *K_I_* in Eqs A10 and A11. The partial derivatives are evaluated at (0,0) yielding the parameters (*a*, *b*, *c*, *d*). Eq. A15 then consist of two first order linear equations governing the dynamic behavior of the non-linear system close to (0,0). Define the parameters β = *a* + *d* and γ = *ad* − *bc*; the eigenvalues λ_1,2_ of the linear system satisfy λ^2^ − βλ + γ =0 with solution

(A16) $$\lambda_{1,2}=\frac{1}{2}\left(\beta \pm \sqrt{\beta^{2}-4\gamma }\right)$$

We are mainly interested in oscillatory solutions about the critical point (0,0); that is, stable limit cycle solutions. These are expected when (0,0) is an *unstable* spiral, which occurs when β > 0 and β^2^ < 4γ. By contrast, stable spirals result in damped oscillations. Saddle points and nodes result in non-oscillatory solutions (stable or unstable) that are of minimal interest here.

Consider the following example with *E*_0_ = *I*_0_. The critical point (*E*_0_, *I*_0_) is unstable if the following condition is met

(A17) $$w_{EE}>\frac{A+1}{AE_{0}\left(1-2E_{0}\right)}\;0<E_{0}<\frac{1}{2}$$

Note that $A=\frac{\tau_{I}}{\tau_{E}}$ so if inhibitory time constants are much shorter than excitatory time constants, larger values of *w_EE_* are required to produce linear instability. For the physiologically interesting range 0.1 < *E*_0_ < 0.4 and *A* = 1, all *w_EE_* > 25 cause the fixed point to be unstable.

For the case *E*_0_ = *I*_0_ and *A* = 1, the fixed point is a spiral if

(A18) $$w_{EE}<2\sqrt{w_{IE}w_{EI}}$$

By combining Eqs A17 and A18 we find the necessary (but possibly not sufficient) conditions for a stable limit cycle about (*E*_0_, *E*_0_) when *A* = 1, that is

(A19) $$\frac{2}{E_{0}\left(1-2E_{0}\right)}<w_{EE}<2\sqrt{w_{IE}w_{EI}}$$

If this condition is met, the corresponding spiral frequency is

(A20) $$\omega_{spiral}=N\left(E_{0}\right)\sqrt{4w_{IE}w_{EI}-w_{EE}^{2}}$$

Here the numerical factor lies in the range 0.00790 < *N*(*E*_0_) < 0.0294 if 0.1 < *E*_0_ < 0.4. An *unstable* spiral point at (*E*_0_, *I*_0_) suggests a likely *stable limit cycle*, but the limit cycle frequency will not generally equal the (linear) spiral frequency. If *w_EE_* exceeds the upper limit in Eq. A19 an unstable node or saddle point will occur. In this case the solutions *X_E_*(*t*), *X_I_*(*t*) are likely to grow beyond physiologically realistic ranges, implying that the basic WC equations are no longer valid.

## References

[B1] Borisyuk R. Denham M. Hoppensteadt F. Kazanovich Y. Vinogradova O. (2000). An oscillatory neural network model of sparse distributed memory and novelty detection. BioSystems 58, 265–272 10.1016/S0303-2647(00)00131-3 11164655

[B2] Bridwell D. A. Srinivasan R. (2012). Distinct attention networks for feature enhancement and suppression in vision. Psychol. Sci. 23, 1151–1158 10.1177/0956797612440099 22923337

[B3] Bush P. Sejnowski T. (1996). Inhibition synchronizes sparsely connected cortical neurons within and between columns in realistic network models. J. Comput. Neurosci. 3, 91–110 10.1007/BF00160806 8840227

[B4] Buzsaki G. Draguhn A. (2004). Neuronal oscillations in cortical networks. Science 304, 1926–1929 10.1126/science.1099745 15218136

[B5] Campbell S. Wang D. (1996). Synchronization and desynchronization in a network of locally coupled Wilson–Cowan oscillators. IEEE Trans. Neural Netw. 7, 541–554 10.1109/72.501714 18263453

[B6] Canolty R. T. Ganguly K. Kennerley S. W. Cadieu C. F. Koepsell K. Wallis J. D. (2010). Oscillatory phase coupling coordinates anatomically dispersed functional cell assemblies. Proc. Natl. Acad. Sci. U.S.A. 107, 17356–17361 10.1073/pnas.1008306107 20855620 PMC2951408

[B7] Canolty R. T. Knight R. T. (2010). The functional role of cross-frequency coupling. Trends Cogn. Sci. (Regul. Ed.) 14, 506–515 10.1016/j.tics.2010.09.001 20932795 PMC3359652

[B8] Chuhma N. Zhang H. Masson J. Zhuang X. Sulzer D. Hen R. (2004). Dopamine neurons mediate a fast excitatory signal via their glutamatergic synapses. J. Neurosci. 24, 972–981 10.1523/JNEUROSCI.4317-03.2004 14749442 PMC6729804

[B9] Demiralp T. Bayraktaroglu Z. Lenz D. Junge S. Busch N. A. Maess B. (2007). Gamma amplitudes are coupled to theta phase in human EEG during visual perception. Int. J. Psychophysiol. 64, 24–30 10.1016/j.ijpsycho.2006.07.005 16956685

[B10] Ding J. Sperling G. Srinivasan R. (2006). Attentional modulation of SSVEP power depends on the network tagged by the flicker frequency. Cereb. Cortex 16, 1016–1029 10.1093/cercor/bhj044 16221931 PMC1880883

[B11] Dustman R. E. Beck E. C. (1965). Phase of Alpha Brain Waves. Electroencephalogr. Clin. Neurophysiol. 18, 433–440 10.1016/0013-4694(65)90123-9 14276036

[B12] Engel A. K. Fries P. Singer W. (2001). Dynamic predictions: oscillations and synchrony in top-down processing. Nat. Rev. Neurosci. 2, 704–716 10.1038/35094565 11584308

[B13] Engel A. K. Singer W. (2001). Temporal binding and the neural correlates of sensory awareness. Trends Cogn. Sci. (Regul. Ed.) 5, 16–25 10.1016/S1364-6613(00)01568-0 11164732

[B14] Fellous J. M. Linster C. (1998). Computational models of neuromodulation. Neural Comput. 10, 771–805 10.1162/089976698300017476 9573404

[B15] Fries P. Nikolic D. Singer W. (2007). The gamma cycle. Trends Neurosci. 30, 309–316 10.1016/j.tins.2007.05.005 17555828

[B16] Goldman-Rakic P. S. Lidow M. S. Gallager D. W. (1990). Overlap of dopaminergic, adrenergic, and serotoninergic receptors and complementarity of their subtypes in primate prefrontal cortex. J. Neurosci. 10, 2125–2138 2165520 10.1523/JNEUROSCI.10-07-02125.1990 PMC6570394

[B17] Gruber W. R. Klimesch W. Sauseng P. Doppelmayr M. (2005). Alpha phase synchronization predicts P1 and N1 latency and amplitude size. Cereb. Cortex 15, 371–377 10.1093/cercor/bhh139 15749980

[B18] Haig A. R. Gordon E. (1998). Prestimulus EEG alpha phase synchronicity influences N100 amplitude and reaction time. Psychophysiology 35, 591–595 10.1017/S0048577298970512 9715102

[B19] Hanslmayr S. Aslan A. Staudigl T. Klimesch W. Herrmann C. S. Bauml K. H. (2007). Prestimulus oscillations predict visual perception performance between and within subjects. Neuroimage 37, 1465–1473 10.1016/j.neuroimage.2007.07.011 17706433

[B20] Izhikevich E. M. (2006). Polychronization: computation with spikes. Neural. Comput. 18, 245–282 10.1162/089976606775093882 16378515

[B21] Izhikevich E. M. Edelman G. M. (2008). Large-scale model of mammalian thalamocortical systems. Proc. Natl. Acad. Sci. U.S.A. 105, 3593–3598 10.1073/pnas.0712231105 18292226 PMC2265160

[B22] Jansen B. H. Brandt M. E. (1991). The effect of the phase of prestimulus alpha activity on the averaged visual evoked response. Electroencephalogr. Clin. Neurophysiol. 80, 241–250 10.1016/0168-5597(91)90107-9 1713834

[B23] Jensen O. Colgin L. L. (2007). Cross-frequency coupling between neuronal oscillations. Trends Cogn. Sci. (Regul. Ed.) 11, 267–269 10.1016/j.tics.2007.05.003 17548233

[B24] Jirsa V. K. Haken H. (1996). Field theory of electromagnetic brain activity. Phys. Rev. Lett. 77, 960–963 10.1103/PhysRevLett.77.960 10062950

[B25] Jirsa V. K. Haken H. (1997). A derivation of a macroscopic field theory of the brain from the quasi-microscopic neural dynamics. Physica D 99, 503–526 10.1016/S0167-2789(96)00166-2

[B26] Koch C. Rapp M. Segev I. (1996). A brief history of time (constants). Cereb. Cortex 6, 93–101 10.1093/cercor/6.2.93 8670642

[B27] Lamme V. A. Roelfsema P. R. (2000). The distinct modes of vision offered by feedforward and recurrent processing. Trends Neurosci. 23, 571–579 10.1016/S0166-2236(00)01657-X 11074267

[B28] Liley D. T. J. Cadusch P. J. Wright J. J. (1999). A continuum theory of electrocortical activity. Neurocomputing 26, 795–800 10.1016/S0925-2312(98)00149-0

[B29] Lisman J. Buzsaki G. (2008). A neural coding scheme formed by the combined function of gamma and theta oscillations. Schizophr. Bull. 34, 974–980 10.1093/schbul/sbn060 18559405 PMC2518638

[B30] Lumer E. D. Edelman G. M. Tononi G. (1997). Neural dynamics in a model of the thalamocortical system. Cereb. Cortex 7, 207–227 10.1093/cercor/7.3.228 9143442

[B31] Menon V. Freeman W. J. Cutillo B. A. Desmond J. E. Ward M. F. Bressler S. L. (1996). Spatio-temporal correlations in human gamma band electrocorticograms. Electroencephalogr. Clin. Neurophysiol. 98, 89–102 10.1016/0013-4694(95)00206-5 8598178

[B32] Mountcastle V. B. (1997). Perceptual Neuroscience: The Cerebral Cortex. Cambridge: Harvard University Press

[B33] Nunez P. L. (1974). The brain wave equation: a model for the EEG. Math. Biosci. 21, 279–297 10.1016/0025-5564(74)90020-0

[B34] Nunez P. L. (1981). Electric Fields of the Brain: The Neurophysics of EEG. New York: Oxford University Press

[B35] Nunez P. L. (1995). Neocortical Dynamics and Human EEG Rhythms. New York: Oxford University Press

[B36] Nunez P. L. (2000). Toward a quantitative description of large-scale neocortical dynamic function and EEG. Behav. Brain Sci. 23, 371–398; discussion 399–437. 10.1017/S0140525X00003253 11301576

[B37] Nunez P. L. Srinivasan R. (2006). Electric Fields of the Brain: The Neurophysics of EEG, 2nd Edn New York: Oxford University Press

[B38] Nunez P. L. Wingeier B. M. Silberstein R. B. (2001). Spatial-temporal structures of human alpha rhythms: theory, microcurrent sources, multiscale measurements, and global binding of local networks. Hum. Brain Mapp. 13, 125–164 10.1002/hbm.1030 11376500 PMC6872048

[B39] Pinotsis D. A. Hansen E. Friston K. J. Jirsa V. K. (2013). Anatomical connectivity and the resting state activity of large cortical networks. Neuroimage 65, 127–138 10.1016/j.neuroimage.2012.10.016 23085498 PMC3520011

[B40] Pinotsis D. A. Schwarzkopf D. S. Litvak V. Rees G. Barnes G. Friston K. J. (2012). Dynamic causal modelling of lateral interactions in the visual cortex. Neuroimage 66C, 563–576 23128079 10.1016/j.neuroimage.2012.10.078 PMC3547173

[B41] Robinson P. A. Rennie C. J. Wright J. J. (1997). Propagation and stability of waves of electrical activity in the cerebral cortex. Phys. Rev. E 55, 826–840 10.1103/PhysRevE.56.826

[B42] Romei V. Rihs T. Brodbeck V. Thut G. (2008). Resting electroencephalogram alpha-power over posterior sites indexes baseline visual cortex excitability. Neuroreport 19, 203–208 10.1097/WNR.0b013e3282f454c4 18185109

[B43] Sarnthein J. Petsche H. Rappelsberger P. Shaw G. L. Von Stein A. (1998). Synchronization between prefrontal and posterior association cortex during human working memory. Proc. Natl. Acad. Sci. U.S.A. 95, 7092–7096 10.1073/pnas.95.12.7092 9618544 PMC22750

[B44] Sarter M. Bruno J. P. (1997). Cognitive functions of cortical acetylcholine: toward a unifying hypothesis. Brain Res. Brain Res. Rev. 23, 28–46 10.1016/S0165-0173(96)00009-4 9063585

[B45] Sauseng P. Klimesch W. Doppelmayr M. Pecherstorfer T. Freunberger R. Hanslmayr S. (2005). EEG alpha synchronization and functional coupling during top-down processing in a working memory task. Hum. Brain Mapp. 26, 148–155 10.1002/hbm.20150 15929084 PMC6871735

[B46] Schalk G. Leuthardt E. C. (2011). Brain-computer interfaces using electrocorticographic signals. IEEE Rev. Biomed. Eng. 4, 140–154 10.1109/RBME.2011.2172408 22273796

[B47] Scheffzuk C. Kukushka V. I. Vyssotski A. L. Draguhn A. Tort A. B. Brankack J. (2011). Selective coupling between theta phase and neocortical fast gamma oscillations during REM-sleep in mice. PLoS ONE 6:e28489 10.1371/journal.pone.0028489 22163023 PMC3230633

[B48] Srinivasan R. Bibi F. A. Nunez P. L. (2006). Steady-state visual evoked potentials: distributed local sources and wave-like dynamics are sensitive to flicker frequency. Brain Topogr. 18, 167–187 10.1007/s10548-006-0267-4 16544207 PMC1995016

[B49] Thorpe S. D’zmura M. Srinivasan R. (2012). Lateralization of frequency-specific networks for covert spatial attention to auditory stimuli. Brain Topogr. 25, 39–54 10.1007/s10548-011-0186-x 21630112 PMC3193902

[B50] Thorpe S. D. S. Garcia J. O. Lee R. R. Huang M. Srinivasan R. (2011). Spatial attention enhances steady-state visual evoked potentials in the gamma band. Int. J. Bioelectromagn. 13, 233–238

[B51] Thut G. Nietzel A. Brandt S. A. Pascual-Leone A. (2006). Alpha-band electroencephalographic activity over occipital cortex indexes visuospatial attention bias and predicts visual target detection. J. Neurosci. 26, 9494–9502 10.1523/JNEUROSCI.0875-06.2006 16971533 PMC6674607

[B52] Traub R. D. Jefferys J. G. Whittington M. A. (1997). Simulation of gamma rhythms in networks of interneurons and pyramidal cells. J. Comput. Neurosci. 4, 141–150 10.1023/A:1008839312043 9154520

[B53] von Stein A. Sarnthein J. (2000). Different frequencies for different scales of cortical integration: from local gamma to long range alpha/theta synchronization. Int. J. Psychophysiol. 38, 301–313 10.1016/S0167-8760(00)00172-0 11102669

[B54] Voytek B. Canolty R. T. Shestyuk A. Crone N. E. Parvizi J. Knight R. T. (2010). Shifts in gamma phase-amplitude coupling frequency from theta to alpha over posterior cortex during visual tasks. Front. Hum. Neurosci. 4:191 10.3389/fnhum.2010.00191 21060716 PMC2972699

[B55] Whittington M. A. Traub R. D. Kopell N. Ermentrout B. Buhl E. H. (2000). Inhibition-based rhythms: experimental and mathematical observations on network dynamics. Int. J. Psychophysiol. 38, 315–336 10.1016/S0167-8760(00)00173-2 11102670

[B56] Wilson H. R. Cowan J. D. (1972). Excitatory and inhibitory interactions in localized populations of model neurons. Biophys. J. 12, 1–24 10.1016/S0006-3495(72)86068-5 4332108 PMC1484078

[B57] Wilson H. R. Cowan J. D. (1973). A mathematical theory of the functional dynamics of cortical and thalamic nervous tissue. Kybernetik 13, 55–80 10.1007/BF00288786 4767470

[B58] Yousif N. A. Denham M. (2005). A population-based model of the nonlinear dynamics of the thalamocortical feedback network displays intrinsic oscillations in the spindling (7–14 Hz) range. Eur. J. Neurosci. 22, 3179–3187 10.1111/j.1460-9568.2005.04496.x 16367784

[B59] Zhang H. Sulzer D. (2004). Frequency-dependent modulation of dopamine release by nicotine. Nat. Neurosci. 7, 581–582 10.1038/nn1350 15146187

